# Segregation distortion: high genetic load suggested by a Chinese shrimp family under high-intensity selection

**DOI:** 10.1038/s41598-020-78389-w

**Published:** 2020-12-11

**Authors:** Qiang Fu, Xianhong Meng, Sheng Luan, Baolong Chen, Jiawang Cao, Xupeng Li, Jie Kong

**Affiliations:** 1 Key Laboratory for Sustainable Utilization of Marine Fisheries Resources, Yellow Sea Fisheries Research Institute, Chinese Academy of Fishery Sciences, Ministry of Agriculture and Rural Affairs, Qingdao, 266071 China; 2 Laboratory for Marine Fisheries Science and Food Production Processes, Qingdao National Laboratory for Marine Science and Technology, Qingdao, 266071 China

**Keywords:** Genetic markers, Genotype

## Abstract

Segregation distortion is a common phenomenon found in most genetic mapping studies and is an important resource to dissect the mechanism of action in gene loci that cause deviation. Marine animals possess high genetic diversity and genomic heterozygosity, they therefore are ideal model organisms to study segregation distortion induced by selection. In the present study, we constructed a full-sib family of *Fenneropenaeus chinensis* and exerted high-intensity selection on 10,000 incipient progenies. 2b-RAD method was employed in remaining 273 individuals to develop genome-wide SNPs for segregating analysis and 41,612 SNPs were developed. 50.77% of 32,229 high-quality representative markers deviated from the expected Mendelian ratio. Results showed that most of these distorted markers (91.57%) were influenced at zygotic level. Heterozygote excess (53.07%) and homozygous deletions (41.96%) may both play an important role, sum of which explained 95.03% of distortion after fertilization. However, further results identified highly probable linkage among deleterious alleles, which may account for a considerable portion of heterozygote excess rather than single locus with heterozygote advantage. Results of this study support a major role of deleterious alleles in genetic load, thus in favor of partial dominance hypothesis. It would also offer necessary recommendations for the formulation of breeding strategy in shrimps.

## Introduction

Segregation distortion (SD) is the deviation of genetic segregation ratios from their expected Mendelian inheritance in a given genotypic class, which was first reported in maize by Mangelsdorf and Jones^1^. It is a common phenomenon that have been widely reported in genetic mapping studies of various species with different types of markers^2–9^. In general, segregation distortion cannot be analyzed by traditional genetic models or theoretical methods^10^, which thus may seriously affect the accuracy of results concluded from genetic maps^11^. However, it is also long accepted that segregation distortion can increase the ratio of heterozygote or heteromorphic chromosome in genetical population, and therefore be identified as a strong evolution force, which has been a hot area of research on theory of natural selection and evolution^12^.

A variety of factors can result in segregation distortion, such as unusual genetic phenomenon including but not limited to chromosomal rearrangement, non-homologous recombination, gene conversion, transposons^13,14^, and non-genetic factors (deviations induced by errors in sequencing or marker genotyping, personal error or sampling error)^15^. But the most common and recognized reasons are always the selection process during gametogenesis, fertilization or prophase development. And it has been proved that the interior elimination criterion on molecular level are some chromosome regions that harbored distorting factors, especially some unfit alleles or genotypes^16,17^. They are also known as genetic load and have long believed to be harmful to population especially in fitness traits^18^. The presence and pattern of segregation distortion in molecular markers, in other words, would be an important proof of genetic load, and provide an effective way to study its genetic mechanism.

The exact genetic mechanism of these unfit loci have been studied for years but scholars still have divergent ideas. The two major hypothesis accounting for the theory of genetic load are partial dominance hypothesis and overdominance hypothesis, which supports a major role for detrimental effects of mutant types and heterozygote advantage, respectively^19^. The two hypothesis have been controversial ever since early twentieth century, as both of them had found direct experimental evidences in various forms of research on the character level^20–23^. In recent studies, partial dominance hypothesis is gradually accepted by more people as the major factor of fitness decline under inbreeding or other forms of genetic purification experiments (generally this is the most common way to expose potential genetic load), by means of modern biotechnology^24^. Whereas the role of overdominance and epistasis are still unclear. The fundamental principles of genetic load cannot be clearly figured out only by analysis on phenotypic level, and molecular basis of elimination criteria can be better understood by studies from genomic level, which are poorly understood in former years but become entirely available benefitted from the burgeoning high-throughput sequencing.

Chinese shrimp, *Fenneropenaeus chinensis*, is one of the most valuable mariculture species in China. Breeding new varieties of *F. chinensis* has always been an urgent demand for China. The Yellow Sea Fisheries Research Institute (Qingdao, China) has initiated a multi-trait selective breeding program for *F. chinensis* ever since 2005. On that basis, the breeding population has been successively selected for 13 years. Recently, a reliable evaluation research of phenotypic and genetic parameters showed that the accumulated realized response was effective in this core breeding population^25^, whereas the inbreeding study in the same population indicated an insusceptible result of inbreeding depression under long-term selection process^26^. This breeding population, in our view, provided good materials to study the way of existence and molecular mechanism of genetic load.

In the present study, we constructed a full-sib family of *F. chinensis* and exerted high-intensity selection in its early life history. It is estimated that amplification of the selective pressure would intensify the elimination of genetic load and lead to severe segregation distortion. Reduced-Representation Sequencing was then employed to develop representative SNPs that evenly covered the whole genome. The objective of this study is therefore to dissect the selection type and pattern of distorted markers from genomic level, and to reveal the severity and genetic mechanism of genetic load. Results of this study would contribute to our understanding of genetic load and offer necessary recommendations for the formulation of breeding strategy in shrimps.

## Results

### Data processing of 2b-RAD sequencing and marker development

A set of 3,787,890,709 reads was output from HiSeq-2500 sequencing system. For the parents, sequencing produced 16.5 and 15.5 million reads, which was 32× and 26× in depth, respectively. 322,153 parent-shared unique tags were generated by clustering parental reads, and 286,345 tags remained after strict filter of low-quality sites. These data formed the high-quality representative reference sites for subsequent genotyping. Sequencing also produced an average of 13.8 million reads per progeny. The sequencing depth for all 273 progenies ranged from 10.2× to 31.5× with an average of 19.8×. Both parents and progenies were sequenced to a sufficient depth that were above the request of high accuracy genotyping^27^. For each representative loci, an average of 255.4 shrimps (93.6% of 273) were genotyped successfully.

In total, 41,612 polymorphic SNP markers were identified from the RAD-typing program. As repetitive sequences or inaccurate results in genotyping may distort our understanding of the segregation law, we further filtered these markers by employing tougher criterion and allowing no more than 2 polymorphic sites exist in each 33-bp read. Finally, 32,229 high-qualified representative SNPs were developed for further segregation analysis.

### Overall performance of segregation distortion

Segregation ratio of parental genotypes was examined first to exclude the possibility of abnormalities in chromosome recombination. The markers we developed showed no significant bias to any parent. It was 1.049:1 between male and female parent in all progenies, calculated by single-parent heterozygous markers. Rate of homozygotes was 45.91% in all loci. There is no obvious bias to homozygote or heterozygote in this population (non-significant).

Among all segregating markers, 15,867 SNPs conformed to the expected Mendelian segregation, whereas approximately half of markers (16,362 SNPs, 50.77%) significantly deviated from it (*P* < 0.05). We suspected that the high-intensity selection had remarkably affected the genetic segregation in this experimental population. Three types of segregating markers exhibited distinguishing proportion and degree of distortion. hk × hk type markers showed a significant higher proportion of distortion compared to the other two types. In addition, most of the distorted markers (77.44%) showed severe deviation (*P* < 0.001, see Table 1).

**Table 1:** Ratios of distorted markers in different types of segregating markers.

Segregation type^#^	Number of markers	Ratios of distortion	With serious distortion*
hk × hk	8,058	77.13%	83.54%
lm × ll	11,938	40.36%	71.01%
nn × np	12,233	43.56%	76.13%
/Total	32,229	50.77%	77.44%

^#^hk × hk represents that markers in both parents are heterozygous; lm × ll represents male parent is heterozygous while the female homozygous; nn × np represents female parent is heterozygous while the male homozygous *The ratio of seriously distorted markers (P < 0.001) to all distorted markers (P < 0.05).

### Gametic and zygotic selection

**Figure 1:**
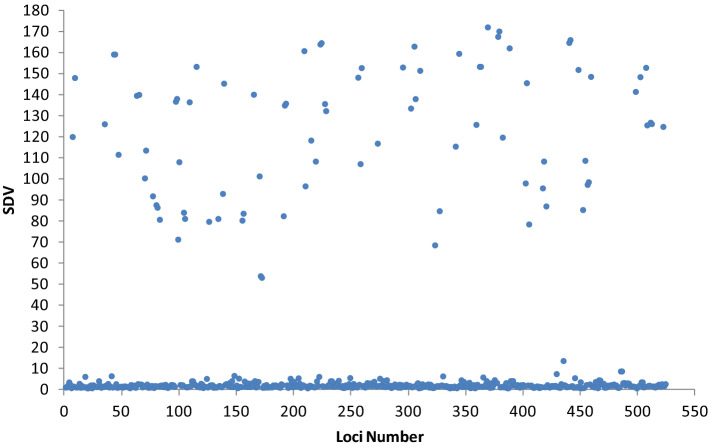
The SDVs of all gametic selection loci. Each dot indicates a marker suffering gametic selection and the ordinate value refers to its segregation distortion value (SDV). Most of them have low SDVs below 5, whereas approximate 15% of the gametic selection loci are distributed in the high SDV region of 60 ~ 180.

In all hk × hk markers that deviated, 91.57% were zygotic selection type (Table 2), which means the leading elimination process occurred after fertilization. The results were consistent with the evident natural elimination in early metamorphosis stages and the imposed artificial selection in larval stage. Nevertheless, there were still 8.43% distorted loci resulting from competition or abortion of the gametes, which suggested that gametic selection do exist before or in fertilization, although the proportion might be very low. The Segregation Distortion Values (SDVs) of 524 gametic selection loci fell into two parts (Fig. 1). Most of them had low SDVs below 5, indicating that selection on these loci were relatively slight, or for other reasons. Whereas, approximate 15% of the gametic selection loci distributed in the high SDV region of 60 ~ 180. In all probability, these markers were linked with gametic genes or gametophyte lethal factors and might suffer severe trial before or in fertilization.

**Table 2:** Test of selection type in deviated hk × hk markers.

*x*_*1*_^2^	*x*_*2*_^2^	Selection Type	Number		Ratio
Non-significant	Significant	Zygotic	4211	5691	91.57%
Significant	Significant	Zygotic	1480		
Significant	Non-significant	Gametic	524		8.43%

### Further analysis of zygotic selection type

The zygotic selection SNPs were further classified into five categories according to the taxonomy described above (for more details see Table 3). The vast majority of zygotic distortion were classed to type 2 and 5 markers, sum of them explained up to 95.03% of total selection. Specifically speaking, type 2 markers that explained 53.07% of zygotic distortion manifested as heterozygote excess, which were probably due to the equivalent elimination of both kinds of homozygote. Type 5 markers that explained 41.96% of zygotic distortion showed homozygous deletion, and they were in all probability owing to the effects of detrimental mutant homozygotes in our understanding. The preliminary results supported an equally important contribution of heterozygote excess and homozygous deletion in elimination of genetic load. Thus, we suspected that overdominance hypothesis (supported by type 2 SNPs) and partial dominance hypothesis (supported by type 5 SNPs) both played an important role in zygotic selection.

**Table 3:** Five classifications of zygotic selection SNPs.

Taxonomy	*AA* : *aa* = 1 : 1					
	√			×		
	( *AA* + *aa* ) : *Aa* = 1:1			( *AA* + *aa* ) : *Aa* = 1:1		
	√	×		√	×	
		(*AA* + *aa*) > *Aa*	(*AA* + *aa* ) < *Aa*		( *AA* + *aa* ) > *Aa*	( *AA* + *aa* ) < *Aa*
Markers	1843	2	3020	221	60	2388
Ratio	–	0.04%	**53.07%**	3.88%	1.05%	**41.96%**
Classify	Normal	Type 1	**Type 2**	Type 3	Type 4	**Type 5**
Possible explanation	–	Heterozygote deficiency	Heterozygote excess	Impact of partial recessive deleterious gene		Homozygous deletion

**Figure 2:**
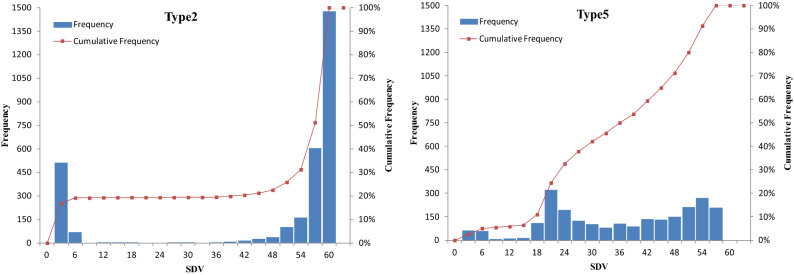
The SDV distribution pattern of deviated markers in type 2 (left) and type 5 (right). Type 2 refers to deviated markers classified as heterozygote excess and type 5 homozygous deletion according to our taxonomy respectively. In both panels, abscissa shows extent of SDVs of these markers. Left ordinate indicates the frequency of markers fallen into each section and is shown in histogram. Right ordinate indicates the cumulative frequency of these markers and is shown as polyline. SDVs of type 2 markers exhibit bimodal distribution. Over 80% of deviated type 2 markers show higher SDV in comparison to the relatively lower SDV of type 5 markers.

However, distribution pattern of SDV showed entirely different characteristics in these two types of deviated markers (Fig. 2). SDVs of type 2 markers exhibited bimodal distribution. Over 80% of deviated type 2 markers showed higher SDV in comparison to the relatively lower SDV of type 5 markers, which was more similar to normal distribution. These results illustrated that the selection effect on most heterozygote advantage loci were more severe than loci that manifested as homozygous deletion.

### Validation of partial dominance markers

**Figure 3:**
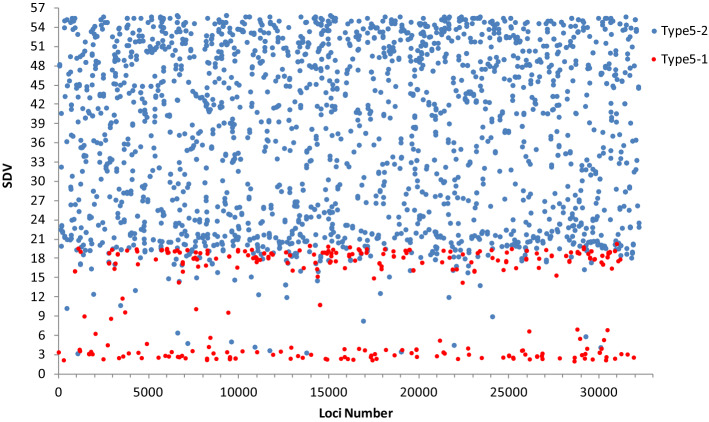
SDVs of type 5 markers with (Type 5-2) and without (Type 5-1) wild-type elimination. 5-1 is represented by red dots and 5-2 by blue dots. The ordinate value refers to segregation distortion values (SDVs) of these markers. The 5-2 markers show higher SDV than those 5-1 markers which have experienced very little wild type elimination.

Type 5 markers that exhibited homozygous deletion were considered as markers supporting partial dominance hypothesis. We went further and tested the ratio of certain homozygote to heterozygote in them, to find out the mutant types suffering elimination. Results showed that only 12.81% of these markers had expectant ratio of wild type to heterozygote (theoretical 1:2) (These markers were defined as 5–1). However, the rest of 87.19% markers (defined as 5–2) showed inexplicable decrease of wild types, although they were no more serious than those in mutant types. It was confirmed by different SDVs showed in Fig. 3. The 5–2 markers showed higher SDV than those 5–1 markers which experienced very little wild type elimination, as a matter of course.

### Classification of single-parent heterozygous loci

**Figure 4:**
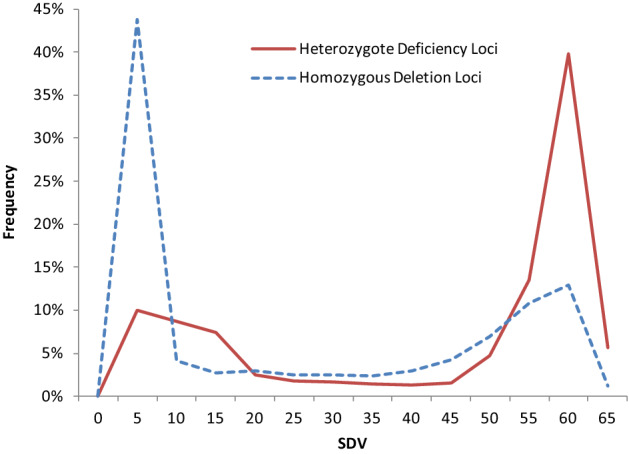
Frequency chart of SDV of two types of deviated markers in single-parent heterozygous loci. Abscissa shows extent of SDVs of these markers. Ordinate indicates the frequency of markers fallen into each section. Solid line represents heterozygote deficiency markers and the imaginary line represents homozygous deletion markers. The heterozygote deficiency loci show more serious distortion than homozygous deletion loci.

lm × ll male heterozygous type and nn × np female heterozygous type SNPs were merged as single-parent heterozygous loci. They were classified based on the decrease of heterozygote or homozygote. Of all 10,147 deviated markers, 55.79% showed heterozygote deficiency and the other 44.21% exhibited homozygous deletion, which might result from the partial dominance effect of detrimental alleles and the superiority of heterozygotes, respectively (Homozygous genotypes in parents were regarded as wild types in this study). This result again supported the combined contribution of partial dominance and overdominance hypothesis in zygotic selection. The distribution pattern of SDV showed more serious deviation in heterozygote deficiency loci compared to homozygous deletion loci (Fig. 4), which illustrated the selection effect on detrimental alleles were more severe than the heterozygote advantage loci. This was in line with our theorized expectations. It is worth noting that the heterozygote deficiency loci in nn × np type showed significant higher rate (65.64%) and more serious deviation (average SDV 44.25) than that in lm × ll type (44.89%; average SDV 34.93), which indicated that the egg was possibly more susceptible to detrimental alleles than sperm.

## Discussion

Segregation distortion is a universal phenomenon in genetic research. Deviations from expected Mendelian segregation ratios in molecular markers have been reported in most marine animals^2,5,8,9,28^ including *Penaeus*^3,4,29^. In most cases, the proportion of deviated markers was not high and they were abandoned in heredity analysis. However, terrible segregation distortion were reported sometimes and they were mainly due to abnormal events in genome structure, such as chromosomal rearrangement or non-homologous recombination in crossing of distantly related individuals^13^. In this study, all systematic factors were carefully avoided. Unusual genetic factors in chromosome level were also unlikely as the parents were derived from a stable population. And above all, segregation ratio test of parental genotypes (1.049:1) and the rate of homozygotes (45.91%) showed no significant bias to any parent or obvious bias to homozygote/heterozygote. This strongly proved that segregation distortion we found in this study, although severe, were not due to errors.

Allele-homologizing populations such as Double Haploid (DH) and Recombinant Inbred Lines (RIL) were the favorite experimental materials for researchers because they can expose the relatively unsuitable alleles or genotypes efficiently^30^. However, strong selection pressure was, although frequently being ignored, another critical factor in this process. Our design was therefore a severe selection process by high-intensity selection in larval stage instead of utilizing an inbreeding population to amplify selection effects of potential detrimental alleles or genotypes. In life history of *F. chinensis*, natural elimination mainly occurred in N, Z, and M larval stages^26^ and the mortality was approximately 40%. Whereas, 300 juvenile shrimps were selected from 10,000 nauplii in our research. The intensity of selection reached up to 3%, and went far beyond that under natural conditions. It was our understanding that this process could immensely amplify the detrimental effects of genetic loads and was the major reason of the novel segregation distortion.

Until the acquisition of full genome sequence variation becomes possible for non-model organisms, molecular markers has been crucial for genetic analysis on genomic level. Genotype-based approaches can gain a better understanding of genetic load through the detection of deviated markers that are closely linked. To further investigate the mechanism of genetic load using genome-wide SNPs, 2b-RAD technology was applied since it featured even and tunable genome coverage and provide reliable, flexible and large scale SNPs^31^. This technique has been successfully applied to mapping studies in marine animals with poorly molecular researches^9,32^. Totally, we developed 32,229 high-qualified representative SNP markers using this method. It was for the first time that large-scale development of SNPs implemented in the genome of *F. chinensis*, which provided valuable sources to reveal the mechanism of genetic load.

The large proportion of deviated markers in experimental population was an indication of the distorting factors abounded in genome, and an evidence of linkage between markers and these distorting factors^16^. This proved that a great deal of genetic loads do exist but the deleterious effect varies a lot, which was in favor of partial dominance hypothesis in a way. The results also validated previous hunches that there was an even larger number of sublethal, subvital or mildly deleterious mutations correlated with fitness traits in marine animals^33^, owing much to the amplification of mild deleterious loci by high-intensity selection process. Further classification (Table 2) proved that zygotic selection was the main selection type for genetic load. The results showed differences in the significant role of gametic selection in some crops^10,34^, but were in accordance with that in most marine animals whose genetic load brought by deleterious alleles had been reported^18,35^.

The two majorities of zygotic selection were heterozygote excess and homozygous deletion (Table 3), suggesting potential heterozygote advantage and selection against detrimental alleles respectively. It seemed that the overdominance and partial dominance hypothesis both played an important role in explanation of genetic load. However, it was unlikely that half of all markers suffered from selection. Segregation distortion was mainly due to Segregation Distortion Loci (SDL) and all markers in the vicinity of SDL would be affected when selection occurred^36,37^. Some studies indicated that an efficient SDL could even affect hundreds Kb of flanking sequence, and the tighter surrounding neutral markers linked, the more severe they distorted^38,39^. This linkage effect in genetic segregation was called "genetic hitch-hiking effect"^39,40^, which can offer an explanation on the surprising proportion of deviated markers. The power of hitch-hiking effect could be easily influenced by recombination rate and selection intensity of the target loci that regulated life cycle or important economic characters in cultured species^41^. An possibly uneven distribution of polymorphic markers^42^ in marker development can exacerbate this effect too. Therefore, the experimental family in our research was a susceptible population to this genetic phenomenon, and the influence of hitch-hiking effects should be taken into account before we drew any conclusion.

**Figure 5:**
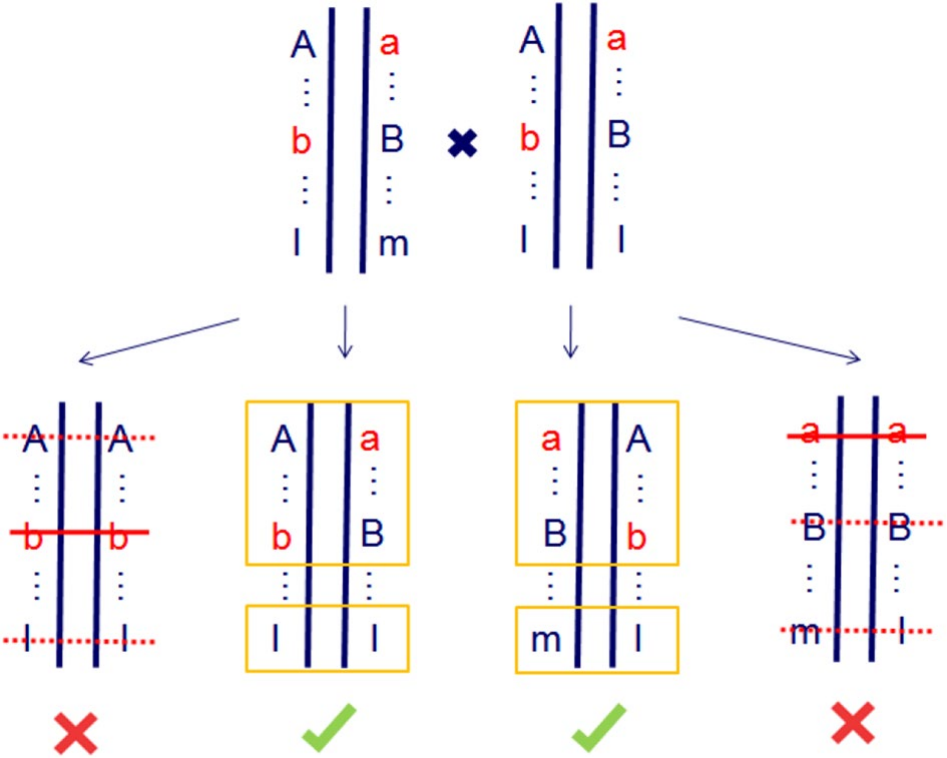
Mode pattern of pseudo-overdominance caused by linkage of detrimental loci and its effect on different types of markers. Three genetic loci are closely linked as haplotypes, and red lowercase indicates partial recessive detrimental alleles. Cross of two heterozygotes have produced four kinds of progenies. Crossed-out lines refer to homozygous detrimental genotypes that will be eliminated, crossed-out imaginary lines indicate neutral genotypes eliminated by hitch-hiking effects. Elimination of homozygous detrimental genotypes leads to a superficial phenomenon of pseudo-overdominance, whereas single-parent heterozygous loci (lm × ll type) do not seem to be affected by hitch-hiking.

In view of the foregoing, we verified again the two major types of zygotic selection markers by means of their SDVs (Fig. 2). From the results, type-2 SNPs that were supported by overdominance hypothesis showed more severe deviation than type-5 SNPs supported by dominant hypothesis. Whereas overdominance loci should be maintained at intermediate frequencies by balancing selection^24^, in other words, elimination of unfit overdominance loci should be no more serious than that of deleterious ones. The contradictory results reminded us that complicated interactions among these loci do exist. The hitch-hiking effects among deleterious alleles may cause pseudo-overdominance, that is, linkage of deleterious alleles lead to the elimination of both homozygotes corporately (Fig. 5). This phenomenon was summarized and warned by Charlesworth and Willis^24^, and could disturb our understanding of single locus with heterozygote advantage. Considering the high selection intensity and the large amount of genetic loci that skewed, linkage among selected loci in a genome region should be usual rather than occasional. In other words, pseudo-overdominance might be extensive in our research.

If so, those severely distorted type-2 markers, which seemingly supported a major role for overdominance but were also readily explicated by deleterious mutations, provided new evidence of pseudo-overdominance. It was worth mentioning that single-parent heterozygous loci do not seem to be affected by hitch-hiking (lm × ll type in Fig. 5). Therefore, the SDVs of heterozygote deficiency loci brought by detrimental alleles showed the expected pattern, which was higher than that of homozygous deletion loci brought by heterozygote advantage (Fig. 4).

As there were no published whole genome sequence of *F. chinensis* or SNP-based genetic maps in previous studies, we cannot study the distribution of distorted markers on genetic maps. Utilization of undistorted markers of this mapping population generated a high-density SNP maps with an average marker density of 0.41 cM (unpublished). However, we failed to add skewed markers to this map as they seriously disturb the normal order of markers. A thorough study were in progress by our group.

**Figure 6:**
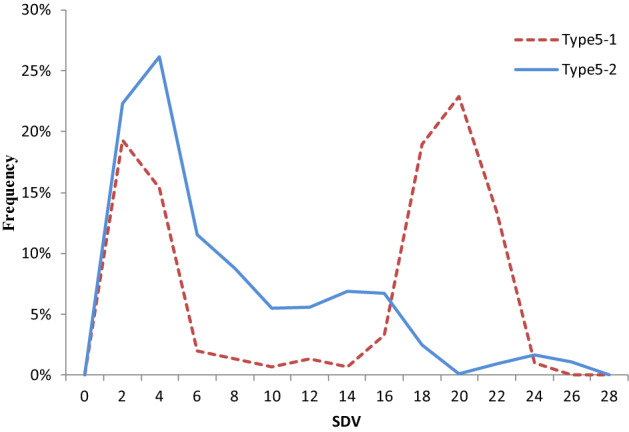
SDV of two homozygotes deviated from 1:1 in type 5-1 and 5-2 markers. Abscissa shows extent of SDVs (deviated from 1:1 of two homozygotes) of these markers. Ordinate indicates the frequency of markers fallen into each section and are shown as polyline. Imaginary line represents type 5-1 markers and solid line represents type 5-2 markers. Deviation between two homozygotes are more serious in 5-1 type .

Logically, the next problem was to find the evidence of pseudo-overdominance—linkage among detrimental loci. Since the eliminations of neutral loci were affected by the genetic distance to SDL, different selection intensity on neutral homozygotes can be considered as an evidence. Fortunately, we found them in analysis of type-5 markers. As shown in 3.5, if selection took place only in mutant types, the ratio of heterozygote and wild type homozygote would be 2:1, verified by *x*^2^ tests, which only happened in 12.81% of type-5 markers (type 5-1). The rest of 87.19% markers (type 5-2) showed a continuous selection background against wild type. In fact, when dividing type-5 markers into two groups by means above, there was a clear distinction in SDV between 5-1 and 5-2 (Fig. 3). The smaller difference between two homozygotes in 5-2 type than that in 5-1 type (Fig. 6) again confirmed this evidence. This provided an inconclusive evidence for the pseudo-overdominance to some extent. Another direct evidence of linkage among detrimental loci was shown in Supplementary Figure S1. Assuming that all zygotic selection markers were in the same linkage group, R^2^ heat map of linkage disequilibrium analysis exhibit tendency of widespread linkage among these loci.

The above evidences point to the fact that deleterious recessive mutations were abundant in *F. chinensis* genome and they played a major role in genetic load. High heterozygosity that resulted in high genetic load has long been reported in marine animals^35,43,44^, which was consistent with our results. There were also gloomy forecasts that an even larger number of sublethal, subvital or mildly deleterious mutations may exist in genome of marine animals^33^. Purging the genetic load was the most common response to this survival pressure in organisms such as crops^45^. However, it seemed that this did not work in shrimps or other marine animals, as the overmuch detrimental loci and the complicated interactions among them we found did not meet the requirements of purging process^46^.

The breeding population of *F. chinensis* had experienced long-term artificial selection, and effective genetic improvements were proved^25^. Genetic loads were supposed to be purged more or less during continuous selection process. However, inbreeding studies in this population showed undiminished inbreeding depression^26^, which means there was no clear signals that detrimental alleles were effectively purged. We speculate that maintaining of high rate of heterozygosity might be the best effective method to withstand high genetic loads. And genetic loads were probably more easily to hide in genome rather than to be purged especially in high heterozygous species.

8 genes were annotated from gametic selection markers (Supplementary Table S1). These genes may play an important role in many biological activities, such as reproduction, metabolism, cell development, differentiation, growth and aging. GO terms and KEGG pathways^48^ from zygotic selection markers with high importance were presented and analyzed in Supplementary Figure S2 and S3. The results indicated that these genes may be involved in many important biological processes and signaling pathways, such as cellular and metabolic process in biological process of GO terms; cell, cell part and organelle in cellular component of GO terms; binding and catalytic activity in molecular function of GO terms; signal transduction, endocrine system, transport and catabolism pathways. However, it is not until the neutral loci affected by hitch-hiking excluded from deviated markers can we find out comparatively accurate genes and pathways potentially involved in the gametic or zygotic selection. We will focus on screening of positive detrimental alleles and their functional study in future.

## Conclusions

Genetic loads in genome of *F. chinensis* were relatively high which could be inferred from the novel segregation distortion performance of a full-sib family under high-intensity selection. Judging from the equally important contribution of heterozygote excess and homozygous deletion in zygotic selection, partial dominance and overdominance hypothesis may both play a part in elimination of unfit alleles. Whereas further analysis supported that deleterious recessive alleles were the major reason of genetic load. Linkage between deleterious alleles were speculated from various aspects, which might result in synergistic elimination of both homozygotes, and could be regarded as new evidences of pseudo-overdominance.

## Materials and methods

### Family construction and high-intensity selection

The parent shrimps are derived from the core breeding population of *F. chinensis*, a new variety named “Huanghai No. 2”. In 2005, a base population was established by crossing several germplasm genetic resources of *F. chinensis*, which consisted of two domesticated breeds ("Huanghai No.1", "Jikang 98") and four wild populations (The south coast of the Korean Peninsula population and three different geographic populations in China). This breeding population had been continuously selected for 11 generations by means of pedigree method (The accumulation of inbreeding was strictly controlled in less than 1% increase per generation). It was now kept in the Marine Genetic Breeding Center of Chinese Academy of Fishery Sciences, Qingdao, China, where our studies were also carried out. The brood stocks had been promoted sexual maturity for over 2 months since January before spawning. Full-sib families were built by strict mating of male and female of known pedigree and distinct phenotypic differences following the mating design, with a method of artificial insemination. The inseminated females were then transported to separate 200 L spawning buckets for spawning.

A standardized procedure for family construction was used in larvae rearing and cultivation of juvenile shrimp^25^, except that high-intensity selections were interspersed in certain developmental stages of life history. Fertilized eggs were incubated in separate buckets for 72 h until they were hatched. A sample of 10,000 nauplii from each family was then transported to another 200L larvae-culture tank by artificial selection for subsequent rearing. Hatched larvae experienced three metamorphosis stages (Nauplius stage (N), Zoea stage (Z), Mysis stage (M)), and about half of them survived into Post-larvae stage (P) under natural selection in about three weeks. Artificial selection were conducted for three times during the Post-larvae stage, 2000 larvae in P5, 1000 larvae in P12 and 500 larvae in P19 were remained and transported to new tanks for further rearing respectively. The selection criterion of better fitness were stronger swimming ability, higher emergency response capability, better feed-taking capacity (according to the degree of digestive gland contents), and deserved bigger body size. When the mean body-length of each family reached one centimeter, a sample of 300 ultimate Post-larvae with highest fitness was transferred into larger tanks (3 m^3^) for subsequent cultivation. After 4 months, the mean body weight of adult shrimps reached about 9 g (There’s almost no natural elimination during this stage). One family with the largest phenotypic variance (indicating relatively high genetic diversity) was singled out and applied to further molecular analysis.

### Library construction and sequencing

A random sample of 273 progenies and two parents of the chosen family were gathered for further high-throughput sequencing. Sequencing strategy in this study was a simple and flexible method known as 2b-RAD, which was a typical Reduced-Representation Sequencing method and became very popular these years owing to its effective cost, high accuracy of genotyping and high representativeness of the whole genome^31^. For all 275 adult shrimps, the muscle was dissected, fixed with 95% alcohol and stored at − 80 °C. Genomic DNA was extracted using TIANamp Marine Animals DNA Kit (TianGen, Beijing). We followed the protocol of Wang et al^31^ to construct standard sequencing libraries using the Type IIB restriction enzyme BsaXI and the original adaptors without any selective base in the terminal 3-bp positions. Therefore, the libraries of 33 bp tags contained almost all recognition sites of BsaXI in the *F. chinensis* genome, which could develop as much markers as possible. Each library contained an individual-specific barcode incorporated during the library preparation to facilitate the pooling of all samples. All libraries were then pooled together as planed and applied to an Illumina Hiseq2500-V2 for single-end sequencing (1 × 50 bp). In consideration of the de novo genotyping strategy and the requirement of establishing high quality reference, libraries of two parents were given extra sequencing depth (1.5-fold higher than ordinary).

### Sequence data processing and genotyping

Raw reads were first filtered to remove adaptors, terminal 3-bp ligation sites, reads with no restriction sites or containing ambiguous base calls (N), reads of inferior quality (over 10 bp homopolymer regions or more than 5 positions with quality score under 10) and mitochondrial origins. The remaining high-quality reads could be applied to subsequent genotyping. As there was no published complete genome sequences of *F. chinensis* until now, we employed the de novo genotyping strategy using the RAD-typing program v1.0^32^. Briefly, pre-processed reads of two parents were combined together and assembled into exactly matched "allele" clusters and "locus" clusters that allowed certain mismatches, successively. Collection of consensus sequences from all "locus" clusters comprised the original parent-shared representative reference sites. The rough references were further filtered by excluding sites that either with insufficient sequencing depth or with depth far above expected (which were most likely derived from repetitive genomic regions or false alignment) using an iML algorithm^47^. In this way, the high-quality reference sites were determined and available for subsequent locus genotyping, that is, sequence reads of two parents and 273 progenies were mapped to those sites separately. The most likely genotype was calculated by posterior probabilities and finally determined by a likelihood ratio test^27^. All genotyping process were conducted under optimized default parameters for marine animals^32^. Polymorphic markers that were heterozygous in at least one parent and could be genotyped in at least 80% of the progenies were considered as qualified segregating markers and retained for further segregating analysis.

### Segregation analysis and statistical analysis

Three types of segregating markers were available for analysis: hk × hk parents-heterozygous type, lm × ll male-heterozygous type and nn × np female-heterozygous type, the expected segregation ratio of which were 1:2:1, 1:1 and 1:1, respectively. For each marker, an initial validation was carried out to assess the goodness of fit of observed with the expected Mendelian ratio using the chi-square test. Those markers deviated from the expected Mendelian ratio (*P* < 0.05) composed the database for further analysis.

Due to the potential selection on more genotypes, hk × hk distorted markers may provide us more valuable information and they are therefore our research emphases. Considering a parents-heterozygous marker, with two alleles *A* and *a*, the genotypes of progenies in F_1_ family were *AA*, *Aa* or *aa*, as expected Mendelian proportions 1:2:1. To determine the types of selection, that is, the impact phase of selection effect, two successive chi-square tests were utilized according to the formulas of Lorieux et al^11^. By checking the balanced frequency of two alleles (*p* = *q*, *x*_*1*_^2^) and the distribution of different genotype frequencies (*p*^2^:2*pq*:*q*^2^, *x*_*2*_^2^), the gametic and/or zygotic selection types would be confirmed:

$${x}_{1}^{2}=\frac{{(2np-n)}^{2}+{(2nq-n)}^{2}}{n}$$

$${x}_{2}^{2}=\frac{{({n}_{AA}-n{p}^{2})}^{2}}{n{p}^{2}}+\frac{{({n}_{Aa}-2npq)}^{2}}{2npq}+\frac{{({n}_{aa}-n{q}^{2})}^{2}}{n{q}^{2}}$$

where *p* = frequency of allele *A*; *q* = frequency of allele *a*; *n* = total sample capacity that genotyped; *n*_*AA*_, *n*_*Aa*_, *n*_*aa*_ represented the number of three genotypes, respectively.

For the zygotic selection markers, it is important to excavate the eliminated genotypes, as distinct elimination pattern indicated distinguishing mechanism. We developed a classification method by a series of assessments on the ratio of three genotypes in which way the eliminative types could be inferred. Ratio test was first utilized between *AA* and *aa* to validate whether they segregated in accordance with 1:1. A test between homozygotes (sum of *AA* and *aa*) and heterozygote was conducted subsequently using the same method. According to the different ratios of three genotypes inferred from each case, the types of elimination were classified into five categories defined as: (1) Heterozygote deficiency; (2) Heterozygote excess; (3) Homozygote deficiency together with slight heterozygote deficiency; (4) Homozygote deficiency together with heterozygote deficiency; (5) Certain homozygous deletion (for more details see Table 3).

The severity of deviation was another important indicator of segregation distortion. It was calculated by chi-square test and shown as *P* value. The *P* value of each distorted marker was converted to its natural logarithm to guarantee the normal distribution. The normalized *P* value was defined as Segregation Distortion Value (SDV) in this study. SDV represented the severity of deviation in each marker, that is, higher SDV in keeping with lower *P* value indicated more serious distortion. Significance level was set to *P* < 0.05 for a distorted marker, and the threshold value of a seriously distorted marker was *P* < 0.001. Linkage disequilibrium between pairs of markers were analyzed by PopLDdecay software (v3.40) and shown in the form of R^2^.

Short sequence tags (27 bp) were first mapped to a survey sequencing database of Chinese shrimp (Soap V2.21, -M 4 -r 0 -v 2), which could be served as mediums for 2b-RAD tags to identify genes surrounding them. The correlated scaffolds were then associated with public genomic resources, Nr、Swissprot、KOG、GO and KEGG^48^. Diamond software were used for Blast (e-value < 1e^−5^). KOBAS and Blast2GO software were used for KEGG and GO annotations. In this step, gametic and zygotic selection loci were analyzed separately.

## Supplementary information

Supplementary Figures

Supplementary Table S1

Supplementary Table S2

Supplementary Table S3

## Data Availability

The detailed information of sequencing and genotyping for the 275 samples were shown in Supplementary Table S2 and Table S3.
