# Supplementary material for: Segregation distortion: high genetic load suggested by a Chinese shrimp family under high-intensity selection
Source: Sci Rep. 2020 Dec 11;10:21820. doi: 10.1038/s41598-020-78389-w (PMC7732831; doi:10.1038/s41598-020-78389-w)
Supplement: Supplementary file 1 — Supplementary Figures [file 41598_2020_78389_MOESM1_ESM.docx]

**Supplementary Figures**

**Title：**Segregation distortion：High genetic load suggested by a Chinese shrimp family under high-intensity selection

**Authors：**Qiang Fu^1,2^, Xianhong Meng^1,2^, Sheng Luan^1,2^, Baolong Chen^1,2^, Jiawang Cao^1,2^, Xupeng Li^1,2^ & Jie Kong^1,2,*^

**Affiliation：**^1^Key Laboratory for Sustainable Utilization of Marine Fisheries Resources, Yellow Sea Fisheries Research Institute, Chinese Academy of Fishery Sciences, Ministry of Agriculture and Rural Affairs. Qingdao, 266071, China;

^2^Laboratory for Marine Fisheries Science and Food Production Processes, Qingdao National Laboratory for Marine Science and Technology. Qingdao, 266071, China.

**^*^**Correspondence and requests for materials should be addressed to J.K. (Email: kongjie@ysfri.ac.cn)


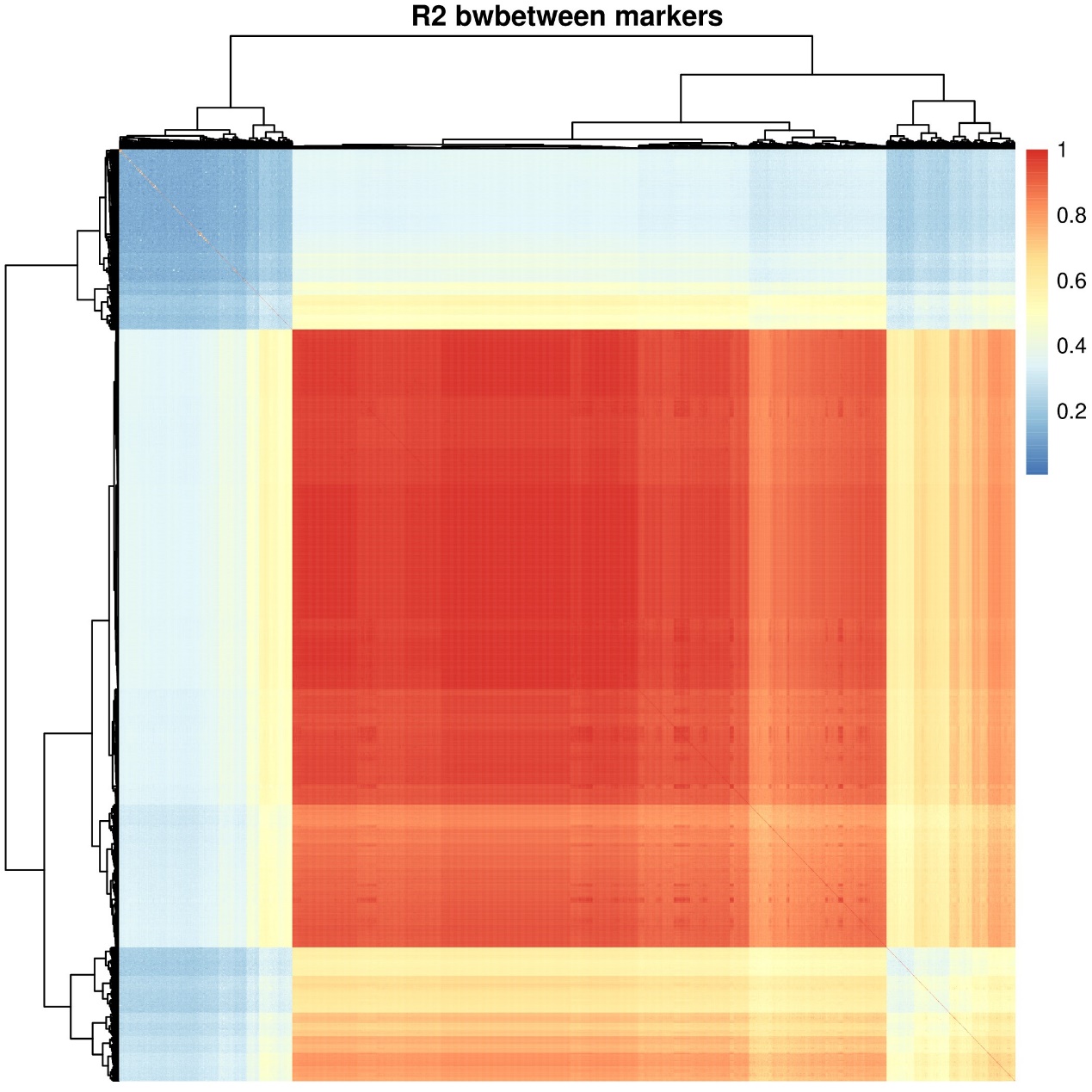


Supplementary Figure S1. R^2^ heatmap of linkage disequilibrium in zygotic selection markers


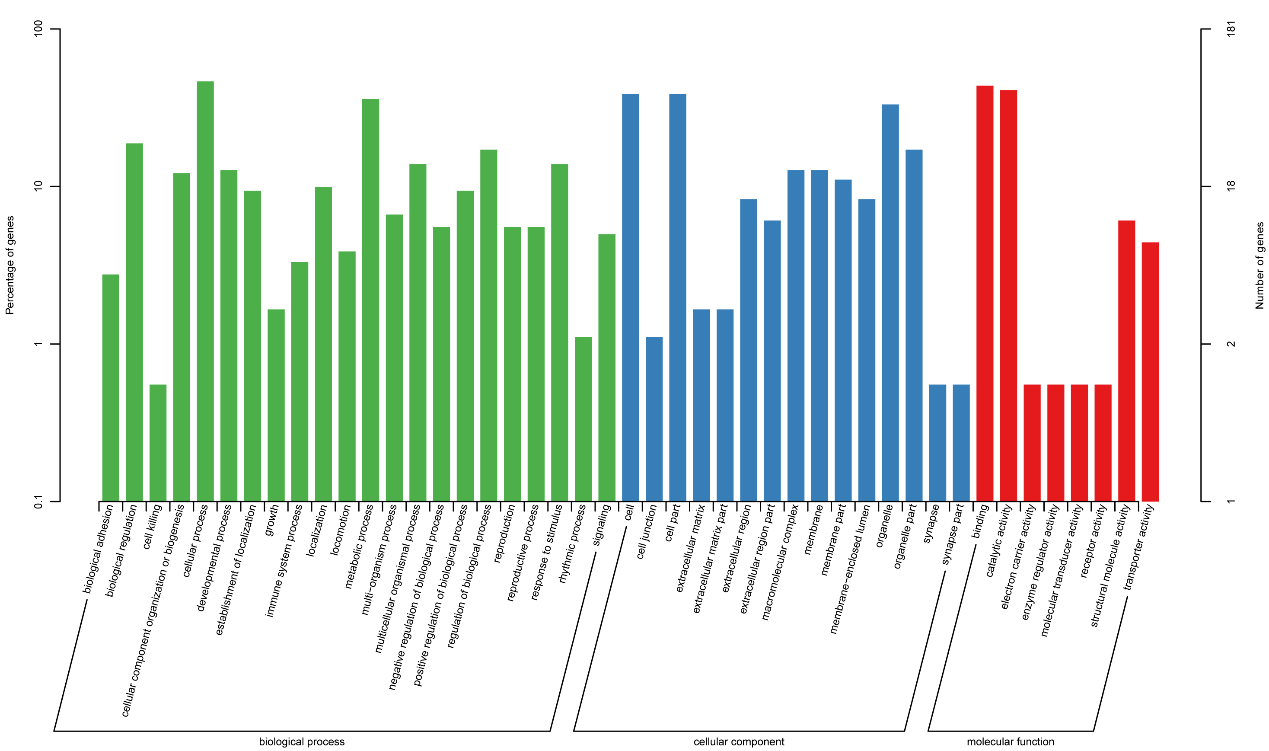


Supplementary Figure S2. Statistics of gene number in GO terms classification from zygotic selection markers


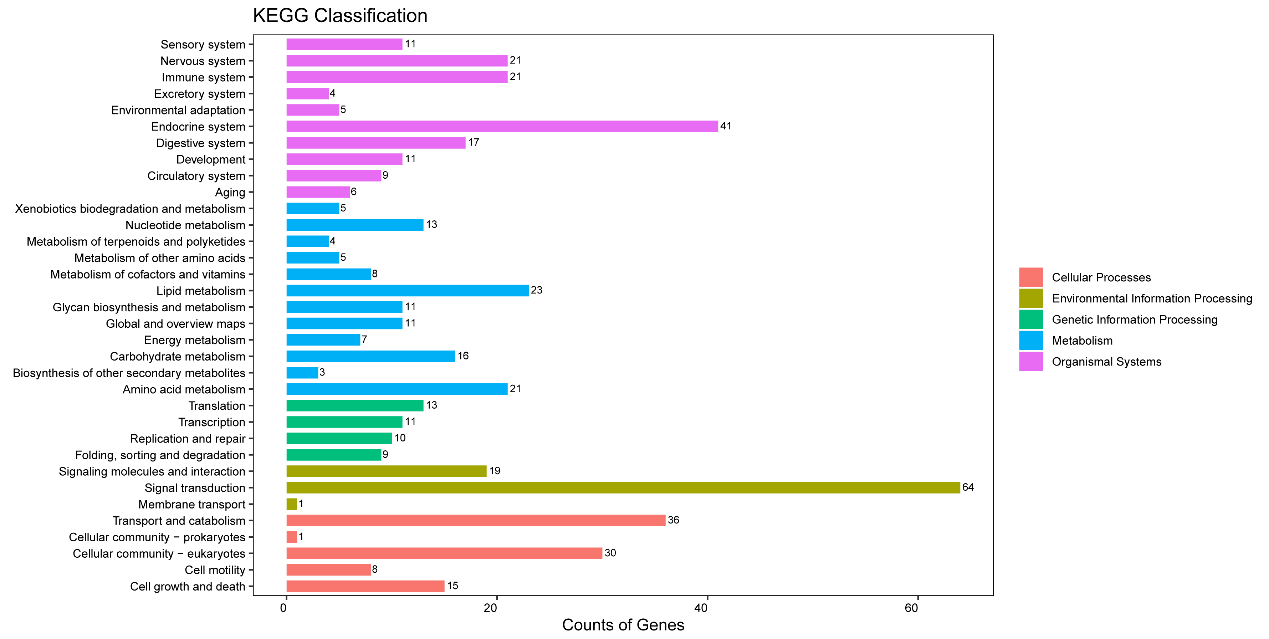


Supplementary Figure S3. Statistics of counts of gene in KEGG classification from zygotic selection markers
